# Upregulation of sphingosine-1-phosphate receptor 3 on fibroblast-like synoviocytes is associated with the development of collagen-induced arthritis via increased interleukin-6 production

**DOI:** 10.1371/journal.pone.0218090

**Published:** 2019-06-07

**Authors:** Takuya Inoue, Masataka Kohno, Hidetake Nagahara, Ken Murakami, Tomoya Sagawa, Akiko Kasahara, Shunya Kaneshita, Takashi Kida, Kazuki Fujioka, Makoto Wada, Hiroshi Nakada, Timothy Hla, Yutaka Kawahito

**Affiliations:** 1 Inflammation and Immunology, Graduate School of Medical Science, Kyoto Prefectural University of Medicine, Kyoto, Japan; 2 Department of Molecular Biosciences, Faculty of Life Sciences, Kyoto Sangyo University, Kyoto, Japan; 3 Department of Surgery, Harvard Medical School, Boston, Massachusetts, United States of America; Keio University, JAPAN

## Abstract

**Background:**

Sphingosine-1-phosphate receptor 3 (S1P_3_) is one of five receptors for sphingosine-1-phosphate (S1P). S1P/S1P_3_ signaling is involved in numerous physiological and pathological processes including bone metabolism, sepsis, cancer, and immunity. In rheumatoid arthritis (RA), fibroblast-like synoviocytes (FLSs) are activated by several factors and promote abundant proinflammatory cytokine production and bone destruction. The aim of this study was to investigate whether S1P_3_ is associated with the development of autoimmune arthritis and the pathogenic function of FLSs.

**Methods:**

Wild-type (WT) and S1P_3_ knockout (S1P_3_-KO) collagen-induced arthritis (CIA) mice were evaluated with respect to clinical and histological disease severity, along with the levels of anti-collagen antibodies and expression of tumor necrosis factor-α (TNFα) and interleukin-6 (IL-6). S1P_3_ expression in the synovium was analyzed by real-time reverse-transcription polymerase chain reaction (RT-PCR) and immunofluorescence staining. FLSs isolated from CIA mice were activated with TNFα and S1P_3_ expression was analyzed by real-time RT-PCR. The role of S1P/S1P_3_ signaling in activated and non-activated FLSs was investigated by measuring cell proliferation and cyto/chemokine production by real-time RT-PCR and/or enzyme-linked immunosorbent assay.

**Results:**

Clinical and histological scores, and synovial IL-6 expression were significantly lower in S1P_3_-KO mice with CIA than in WT mice. Arthritic synovia had higher S1P_3_ expression than intact synovia and FLSs in arthritic joints expressed S1P_3_ *in vivo*. Primary cultured FLSs produced IL-6 in a time-dependent manner in response to S1P stimulation and exhibited higher levels of S1P_3_ expression after activation with TNFα. S1P_3_-induced production of IL-6 and MMP-3 was increased in FLSs pre-activated with TNFα.

**Conclusion:**

In this study, we demonstrated that S1P_3_ expression is associated with the development of autoimmune arthritis via inflammation-induced increases in S1P/S1P_3_ signaling that increase production of IL-6 in FLSs. Inhibition of S1P/S1P_3_ signaling could open the door to the development of new therapies for RA.

## Introduction

Rheumatoid arthritis (RA) is a chronic systemic inflammatory disease that can cause cartilage damage, bone erosion, and joint dysfunction, resulting in irreversible disability [1]. The cause of RA remains mostly unknown, but genetic and environmental factors are involved in its pathogenesis [2]. A recent therapeutic strategy referred to as the “treat-to-target” approach requires assessment of disease activity and modification of management in accordance with such activity [3, 4]. New anti-rheumatic drugs targeting tumor necrosis factor α (TNFα), interleukin-6 (IL-6), and several surface markers expressed on B cells or T cells have been approved in the past two decades. However, about a third of RA patients cannot tolerate or attain disease remission with these agents [5], and novel therapeutic approaches are needed.

Sphingosine-1-phosphate (S1P) is a bioactive lipid mediator involved in several physiological and pathological conditions including autoimmune disease, cardiovascular disease, cancer, sepsis, and bone metabolism [6]. It acts as a ligand for its five receptors, S1P_1–5_, in an autocrine and a paracrine manner, and contributes to cell differentiation, survival, migration, and cytokine/chemokine secretion. Levels of S1P are higher in the synovial fluid of RA patients than in that of osteoarthritis (OA) patients [7], and the expression of S1P_3_ in fibroblast-like synoviocytes (FLSs) from RA patients is upregulated by TNFα *in vitro* [8]. These observations suggest that S1P/S1P_3_ signaling may be involved in the pathogenesis of RA.

The most prominent morphological feature of RA is formation of the pannus, a layer of hyperplastic synovium with a lining mainly composed of activated FLSs, which help initiate and perpetuate the disease. Activated FLSs show increased migratory capacity and invasive potential and produce large amounts of proinflammatory cytokines, chemokines, and matrix-degrading enzymes [9, 10], which contribute to cartilage erosion and bone destruction [11]. FLS activation can also be induced by proinflammatory cytokines such as TNFα, cell-cell contact, or Toll-like receptor ligands [12]. However, it remains unclear whether S1P_3_ is upregulated in these FLSs *in vivo* and whether S1P/S1P_3_ signaling plays a significant role in the pathogenesis of RA.

In this study, we investigated the role of S1P_3_ in the collagen-induced arthritis (CIA) mouse model using S1P_3_ knockout (S1P_3_-KO) mice and primary cultured FLSs. The severity of CIA and levels of cytokine expression in the synovium of wild-type (WT) mice were compared with those in S1P_3_-KO mice; in addition, S1P_3_ expression in FLSs was analyzed. Furthermore, we evaluated expression of S1P_3_ and its effect on production of arthritogenic molecules by TNFα-activated primary FLSs. We demonstrated that S1P_3_ expression contributes to the development of CIA via inflammation-induced upregulation of S1P/S1P_3_ signaling, which increases the production of IL-6 by FLSs.

## Materials and methods

### Mice

S1P_3_-KO (*S1p3*^-/-^) mice on a mixed DBA/1J-129/Sv background were generated as previously described [13, 14]. These mice were backcrossed with *S1p3*^+/+^ DBA/1J mice (Shimizu Laboratory Supplies Co., Kyoto, Japan) for nine generations to obtain *S1p3*^+/-^ DBA/1J mice. The backcrossed *S1p3*^+/-^ DBA/1J mice were then intercrossed to generate *S1p3*^-/-^ DBA/1J mice and *S1p3*^+/+^ littermates, which were then mated with mice of the same genotype, and their descendants were used in experiments. The S1P_3_ genotypes were determined by PCR analysis of genomic DNA isolated from tail biopsy specimens [14]. All mice were provided with standard laboratory pellets and water *ad libitum*, and maintained in an atmosphere of 24 ± 2°C with a 12 hour light/dark cycle under specific pathogen-free conditions in the Animal Resource Facility at Kyoto Sangyo University. Animal experiments were approved by the Kyoto Sango University Committee on Animal Welfare (approval number 2011–07).

### Collagen-induced arthritis model

The induction of arthritis in mice was performed as previously described [15]. Briefly, male S1P_3_-KO or WT DBA/1J mice at 8–12 weeks of age were immunized by intradermal injection at the base of the tail with 100 μg bovine type II collagen (CII; Chondrex, Redmond, Washington, USA) emulsified in 50 μg complete Freund’s adjuvant (Chondrex) on Day 0, followed by a booster injection with 100 μg bovine CII emulsified in 50 μg incomplete Freund’s adjuvant (Chondrex) on Day 21. Mice were sacrificed on Day 42 under anesthesia by peritoneal injection with a combination of 0.3 mg/kg medetomidine, 4 mg/kg midazolam, and 5 mg/kg butorphanol. In the case of excessive weight loss (greater than 20% of the baseline body weight), mice were euthanized.

Arthritis severity was assessed in a blinded manner three times per week from Days 21 to 42. Each paw was scored on a scale of 0–4 according to a previously reported scoring system [16] with some modification, as follows: 0 = normal; 1 = mild, slight swelling at the wrist/ankle joint or individual digits; 2 = moderate, apparent swelling confined to either the wrist/ankle joint or individual digits; 3 = severe swelling extending from the wrist/ankle joint to individual digits; 4 = severe swelling encompassing the wrist/ankle and digits. The arthritis score was recorded as the sum of the scores for individual paws, resulting in a maximum possible score of 16 per mouse.

### Histological assessment

For histological analysis, hindlimbs were removed from sacrificed mice on Day 42 and then fixed in phosphate buffer containing 4% paraformaldehyde (PFA; Wako, Kyoto, Japan) overnight at 4°C, embedded in paraffin, sectioned, and stained with hematoxylin and eosin (H&E) for microscopic assessment. The severity of arthritis in each ankle joint was evaluated based on the degree of synovial inflammation, bone erosion, and cartilage destruction by two scientists blinded with respect to S1P_3_ genotype. Each parameter was graded on a scale of 0–3 as previously described [17].

Immunofluorescent staining with an anti-S1P_3_ antibody was performed on ankle joint sections. Preparation of fresh-frozen sections and immunofluorescence staining were carried out using Kawamoto’s film method [18] with some modifications. Briefly, hindlimbs were fixed in 4% PFA for 3 hours at 4°C, embedded in SCEM cryoembedding compound (Section-Lab, Hiroshima, Japan), attached to adhesive plastic film (Section-Lab), and sectioned to a 5 μm thickness. The specimens were blocked with Blocking One Histo (Nacalai Tesque, Kyoto, Japan) for 10 minutes and then incubated overnight at 4°C with the following primary antibodies: rabbit anti-S1P_3_ antibody, 10 μg/mL (PA5-77744, RRID: AB_2735752; Thermo Fisher Scientific, Waltham, Massachusetts, USA); Syrian hamster anti-podoplanin/gp36 antibody, 8 μg/mL (ab11936, RRID:AB_298718; Abcam, Cambridge, UK); rabbit IgG, 10 μg/mL (GTX35035, RRID:AB_10623175; GeneTex, Irvine, California, USA); or Syrian hamster IgG, 8 μg/mL (ab18426, Abcam). Subsequently, the sections were incubated for an hour at ambient temperature with fluorescein-conjugated secondary antibodies: Alexa Fluor 488-conjugated goat anti-Syrian hamster IgG, 4 μg/mL (ab180063, Abcam); or Alexa Fluor 594-conjugated donkey anti-rabbit IgG, 4 μg/mL (ab150064, RRID:AB_2734146; Abcam). The nuclei were stained with 4',6-diamidino-2-phenylindole (DAPI; Dojindo, Kumamoto, Japan). The sections were washed with phosphate-buffered saline (PBS) between each step. All stained sections were analyzed with a fluorescence microscope (BZ-X710; Keyenence, Osaka, Japan).

### Measurement of serum anti-collagen antibody levels

Blood, collected from CIA mice sacrificed on Day 42, was subjected to serum analysis. Levels of anti-CII IgG_1_ and IgG_2_ were measured using a Mouse Anti-type II Collagen IgG Subtype Antibody ELISA Kit (Chondrex), according to the manufacturer’s instructions.

### RNA extraction from knee joint capsules and quantitative real-time RT-PCR

CIA mice were sacrificed at Day 42. The joint capsules were isolated from the knee joints using a previously reported method [19] and treated with RNAlater RNA stabilization reagent (Qiagen, Hilden, Germany). Isolated joint capsules frozen in liquid nitrogen were disrupted in a freeze mill and homogenized with a QIAshredder (Qiagen). Total RNA was extracted from the homogenate using the RNeasy Plus Mini Kit (Qiagen), and reverse transcription was carried out using the High Capacity cDNA Reverse Transcription Kit (Applied Biosystems/Thermo Fisher Scientific) according to the manufacturer’s instructions. Quantitative real-time RT-PCR was conducted using the StepOne Plus Real-time PCR System (Applied Biosystems) with Thunderbird Probe qPCR Mix (Toyobo, Osaka, Japan) and the following Taqman Gene Expression Assays (Applied Biosystems): *Tnf*α (Mm00443258_m1), *Il-6* (Mm00446191_m1), *S1p3* (Mm00446191_m1), and *Gapdh* (Mm99999915_g1). The expression of target genes relative to the expression of *Gapdh* was quantified using the ΔΔC_T_ method.

### Isolation and culture of fibroblast-like synoviocytes

Murine FLSs were isolated from CIA mice 10 ± 2 days after the onset of arthritis according to previously established protocols with slight modifications [19, 20]. In brief, the knee joint capsules were minced and digested with 400 μg/mL liberase (Roche, Basel, Switzerland) in serum-free Dulbecco’s modified Eagle medium (DMEM; Nacalai Tesque, Kyoto, Japan) at 37°C for 30 minutes. After filtration through a 70 μm nylon cell strainer (Corning, Corning, New York, USA), the filtrate was centrifuged at 1,500 × *g* for 5 minutes at 4°C and resuspended in DMEM supplemented with 10% fetal bovine serum (FBS), 100 U/mL penicillin, and 100 μg/mL streptomycin. The cells were seeded onto 6-well tissue culture plates and cultivated in a humidified incubator (37°C, 5% CO_2_). The medium was changed every 3–4 days. FLSs grown to 80–90% confluence were harvested with 0.25% trypsin and 1 mM EDTA and re-plated at a dilution of 1:4. FLSs at passage 3–4 were used in subsequent experiments.

### Proliferation assays

FLSs pre-cultured overnight at a density of 2.5 × 10^4^ cells/well in 96-well plates were stimulated with S1P (0–5 μM) in DMEM containing 10% FBS for 48 hours. Cell proliferation was quantified using the Cell Counting Kit 8 (Dojindo) according to the manufacturer’s instructions.

### Stimulation of FLSs with S1P and/or TNFα

To investigate the expression of S1P_3_ in activated FLSs, S1P_3_ mRNA in FLSs activated with TNFα was analyzed by real-time RT-PCR. FLSs were seeded at a density of 4 × 10^5^ cells/mL in 96-well plates and incubated for 24 hours. After serum starvation for 3 hours, the cells were incubated in DMEM with 1% FBS containing 10 ng/mL TNFα (PeproTech, Rocky Hill, New Jersey, USA), or vehicle for 3 hours. In some experiments, FLSs were serum-starved overnight, pre-treated with TNFα (10 ng/mL, 8 hours), then stimulated with S1P (5 μM, 3 hours). Total RNA extraction and reverse transcription were carried out with the SuperPrep Cell Lysis and RT Kit for qPCR (Toyobo) according to the manufacturer’s instructions. Quantitative real-time PCR was performed for *Il-6*, *S1p3*, and *Gapdh* as described above.

### Enzyme-linked immunosorbent assays

FLSs were seeded at a density of 4 × 10^5^ cells/mL in 24-well plates and incubated for 24 hours. After overnight serum starvation, the FLSs were stimulated with S1P (5 μM, 8–24 hours). In some experiments, FLSs were pre-treated with TNFα (10 ng/mL, 8 hours) prior to stimulation with S1P (5 μM, 24 hours). The cell culture supernatant was collected and stored at -80°C until analysis. The levels of IL-6, MCP-1 and matrix metalloproteinase 3 (MMP-3) were measured using an IL-6 Mouse ELISA Kit (Invitrogen/Thermo Fisher Scientific), a MCP-1 Mouse ELISA Kit (BioLegend, San Diego, California, USA), and a Mouse Total MMP-3 Quantikine ELISA Kit (R&D systems, Minneapolis, Minnesota, USA), respectively, according to the manufacturer’s instructions.

### Statistical analysis

Data are expressed as the mean ± standard error of the mean (SEM) for parametric data and as the median and interquartile range (IQR) for non-parametric data. Parametric data were compared by unpaired *t*-test, and non-parametric data were analyzed by Mann-Whitney U-test. Comparisons among multiple groups were performed by one-way analysis of variance (ANOVA) followed by Dunnett’s test or a *t*-test with Bonferroni’s correction. Statistical analysis of arthritis scores was performed at Days 36, 38, 40, and 42, when scores were expected to be the highest. The scores were analyzed by *t*-tests with Bonferroni’s correction. P-values < 0.05 were considered statistically significant. All data were analyzed using EZR version 1.37 (Saitama Medical Center, Jichi Medical University, Japan), a graphical user interface for R (The R Foundation for Statistical Computing) [21].

## Results

### Alleviation of collagen-induced arthritis in S1P_3_-KO mice

**Fig 1:**
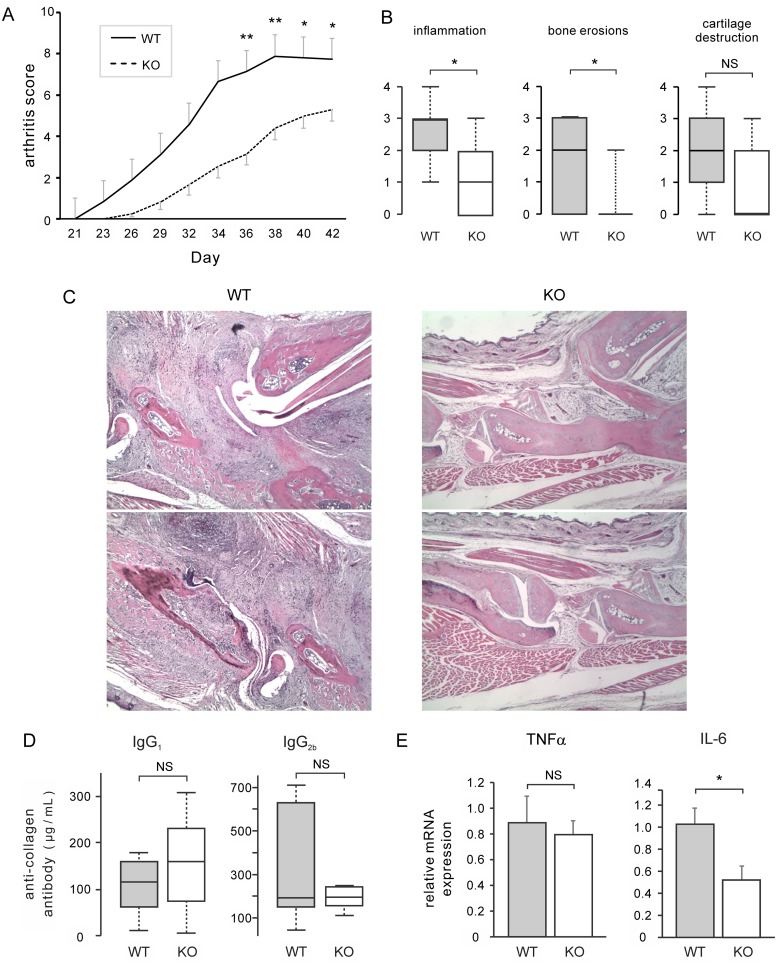
Sphingosine-1-phosphate receptor 3 deficiency attenuates clinical and histological scores and IL-6 expression in collagen-induced arthritis models. (A) Development of collagen-induced arthritis in WT and S1P_3_ knockout (S1P_3_-KO) mice. Arthritis was evaluated three times per week during Days 21–42. Disease severity was measured by assessing the clinical score of all four paws, in which the maximum possible score for an individual mouse was 16. Statistical analysis was performed at Days 36, 38, 40, and 42. Results are presented as the mean ± SEM (n = 20 mice per group) and were analyzed by *t*-test with Bonferroni’s correction. * P < 0.05 and ** P < 0.01 (WT vs. KO mice). (B) Synovial inflammation, bone erosion, and cartilage destruction were examined histologically on H&E-stained sections of ankle joints and assigned scores of 1–4. Results are presented as box-and-whisker plots (n = 20 mice per group) and were analyzed by Mann-Whitney U-test. The middle hash, the box, and the whiskers represent the median, interquartile range (IQR), and 10/90 percentile values respectively. * P < 0.05, NS = not significant. (C) Representative images of H&E-stained ankle joint sections are shown at a magnification of 40×. Left panel: WT specimen assigned a score of 3 for all parameters. Right panel: KO specimen assigned a score of 1 for inflammation and 0 for the other parameters. (D) Anti-CII IgG_1_ and IgG_2_ antibodies in serum from CIA mice were measured in an ELISA. Results are presented as box-and-whisker plots (n = 10 mice per group) and were analyzed by Mann-Whitney U-test. The middle hash, the box, and the whiskers represent the median, IQR, and minimum/maximum values respectively. (E) Expression of TNFα and IL-6 in arthritic synovium was analyzed by real-time RT-PCR. Results are presented as the mean ± SEM (n = 6 mice per group) and were analyzed by *t*-test. * P < 0.05, NS = not significant.

To determine whether S1P_3_ affects arthritis severity, arthritis scores were compared over time between WT and S1P_3_-KO mice with CIA (Fig 1A). The mean arthritis scores were lower in the S1P_3_-KO group than in the WT group at all time points. These differences were significant at Days 36, 38, 40, and 42.

### Attenuated histological scores for arthritis indicators in S1P_3_-KO mice

To confirm the finding of reduced disease severity, histological changes were also compared between the two groups (Fig 1B and 1C). The severity of inflammation and the extent of bone erosion and cartilage destruction were evaluated in H&E-stained ankle joint sections. The S1P_3_-KO group had significantly less synovial inflammation and bone erosion than the WT group. Cartilage destruction tended to be lower in the S1P_3_-KO group, but the difference was not statistically significant.

### Anti-collagen antibody and inflammatory cytokines in S1P_3_-KO mice

Levels of anti-CII IgG_1_ and IgG_2_ antibodies in serum from CIA mice were measured in an ELISA (Fig 1D) and expression of inflammatory cytokines in the synovium was determined by real-time RT-PCR (Fig 1E). Deficiency of S1P_3_ had no significant effect on levels of anti-CII IgG_1_ antibody (WT median 115 μg/mL, IQR 62–160 μg/mL versus S1P_3_-KO median 160 μg/mL, IQR 74–230 μg/mL; p = 0.743) and IgG_2_ antibody (WT median 192.8 μg/mL, IQR 150.5–624.8 μg/mL versus S1P_3_-KO median 196.0 μg/mL, IQR 162.6–237.8 μg/mL; p = 0.662). Expression of IL-6 in S1P_3_-KO CIA mice was significantly lower than that in WT mice, whereas that of TNFα was comparable with that in WT mice.

### Expression of S1P_3_ in arthritic joints

**Fig 2:**
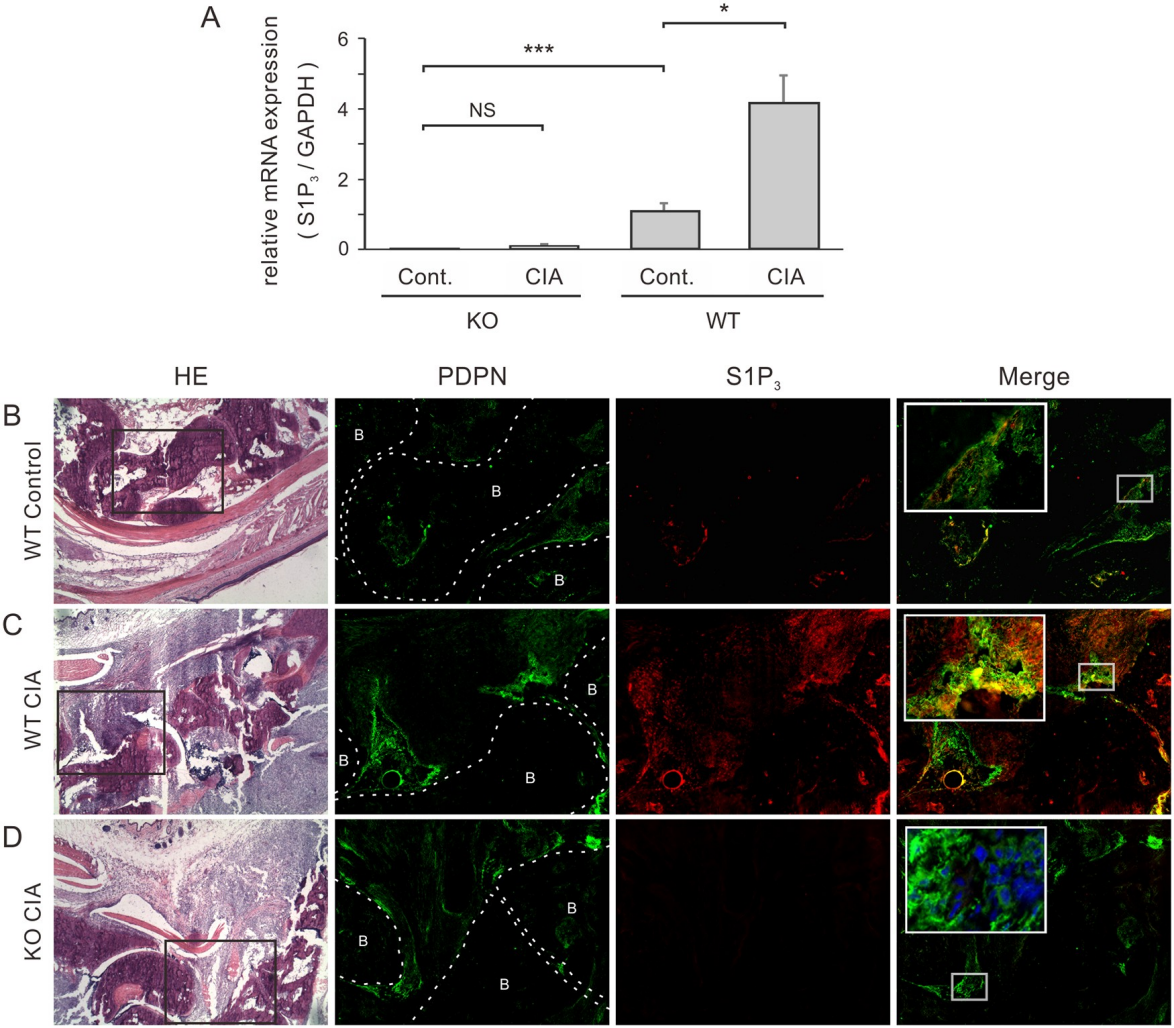
Expression of sphingosine-1-phosphate receptor 3 is upregulated in arthritic joints. (A) S1P_3_ mRNA was quantified in intact and arthritic joint capsules by real-time RT-PCR. Results are presented as the mean ± SEM (n = 4 mice per group) and were analyzed by *t*-test with Bonferroni’s correction. * P < 0.05, ** P < 0.01, *** P < 0.001, NS = not significant. (B–D) H&E staining and double immunofluorescence staining were performed on frozen sections of ankle joints using an anti-S1P_3_ antibody (red) and an anti-PDPN antibody (green), which was used as a marker of activated FLSs. There were few PDPN-positive cells in intact joints of WT mice (B), and these cells were almost completely negative for S1P_3_. There were many more PDPN-positive cells in severely inflamed joints of WT mice (C) and S1P_3_-KO mice (D). Most PDPN-positive FLSs in WT mice, but not those in KO mice, were also positive for S1P_3_ (insets in C and D, respectively). The insets show high-magnification images of the areas indicated by the open squares. Representative images from three mice per group are shown at a magnification of 40× or 100× (insets). B = bone.

Since the expression of S1P receptors is regulated by several types of inflammation, we investigated whether S1P_3_ expression was affected by arthritis. S1P_3_ expression was assessed in arthritic synovia by real-time PCR (Fig 2A) and immunofluorescent staining (Fig 2B–2D). Total RNA was extracted from WT and S1P_3_-KO joint capsules from mice with or without arthritis. Normal WT synovia had higher levels of *S1p3* mRNA than synovia from healthy S1P_3_-KO (Fig 2A). Furthermore, the expression of S1P_3_ was approximately 4-fold higher in arthritic synovia from WT mice.

Next, to determine which cells upregulated S1P_3_ in arthritic joints, we focused on FLSs, which are one of the most abundant cell types in the arthritic synovium. Double immunofluorescence staining was performed on ankle joint sections using an anti-S1P_3_ antibody and an antibody against PDPN, a cell surface marker of FLSs [10] (Fig 2B–2D). Although a limited number of PDPN-positive cells (FLSs) were detected in intact joints from WT mice (Fig 2B, green), they showed little positivity for S1P_3_ (Fig 2B, red). On the other hand, FLSs were abundant in severely affected joints from WT or S1P_3_-KO mice with CIA (Fig 2C and 2D, green), and most of the FLSs in inflamed synovia of WT mice (but not KO mice) were also S1P_3_-positive (Fig 2C).

### Effect of S1P/S1P_3_ signaling on non-activated fibroblast-like synoviocytes

**Fig 3:**
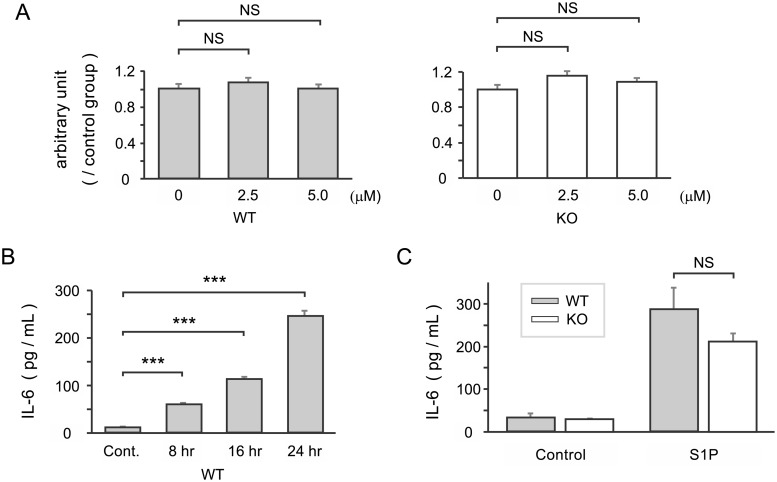
Effect of sphingosine-1-phosphate on cell proliferation and IL-6 production by cultured fibroblast-like synoviocytes. (A) FLSs were cultured for 48 hours with S1P (0, 2.5, and 5.0 μM) and proliferation assays were performed. The results are presented as a ratio with respect to the control group. (B) IL-6 levels in supernatants from WT FLSs cultured with 5 μM S1P for the indicated periods of time were measured in an ELISA. (C) WT and S1P_3_-KO FLSs were incubated with 5 μM S1P for 24 hours, and IL-6 production was compared. Results are presented as the mean ± SEM (n = 4 samples per group) and were analyzed by one-way ANOVA followed by Dunnett’s test (A, B) or a *t*-test (C). * P < 0.05, ** P < 0.01, *** P < 0.001, NS = not significant.

The impact of S1P on cell proliferation and cytokine production was investigated in FLSs. Primary FLSs were cultured in DMEM containing S1P (0, 2.5, or 5 μM) for 48 hours, and the cell numbers were determined using commercial cell counting kits (Fig 3A). The cell numbers did not significantly increase after stimulation with S1P (2.5 or 5 μM).

Next, FLSs were incubated with 5 μM S1P for various periods of time and the levels of IL-6 in the supernatant were measured by ELISA. The secretion of IL-6 increased significantly in a time-dependent manner (Fig 3B). However, there was no significant difference in IL-6 production between the two genotypes (Fig 3C: WT 286.5 ± 50.4 pg/mL versus S1P_3_-KO 211.5 ± 19.5pg/mL; p = 0.215). These results suggested that S1P promoted IL-6 production by FLSs but that S1P_3_ signaling did not play a significant role in this process, at least in non-activated FLSs.

### S1P_3_ expression on FLSs activated with TNFα

**Fig 4:**
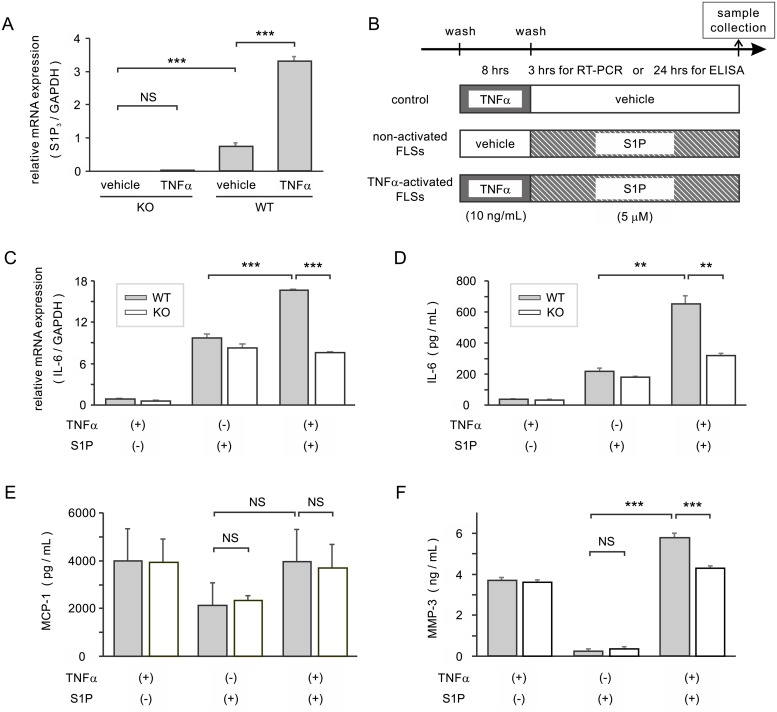
Upregulated sphingosine-1-phosphate receptor 3 and S1P/S1P_3_-induced production of arthritogenic molecules by activated FLSs. (A) S1P_3_ expression in FLSs cultured with TNFα or vehicle was analyzed by real-time RT-PCR. (B) Schematic representation of the following experiments, in which FLSs were stimulated with S1P with or without prior activation by TNFα. (C) Expression of IL-6 by FLSs was analyzed by real-time RT-PCR. (D) The levels of IL-6 in the supernatant were measured in an ELISA. (E, F) The levels of MCP-1 and MMP-3 in the same supernatant were measured in an ELISA. Results are presented as the mean ± SEM (n = 4 samples per group) and were analyzed by ANOVA followed by a *t*-test with Bonferroni’s correction. * P < 0.05, ** P < 0.01, *** P < 0.001, NS = not significant.

S1P_3_ expression was upregulated on FLSs in inflamed synovia (Fig 2C), and it has been shown that FLSs are activated by TNFα, one of the most abundant cytokines implicated in RA, *in vitro*. To investigate the effect of TNFα on S1P_3_ expression in cultured FLSs, total RNA was extracted from primary FLSs cultured with TNFα (10 ng/mL) for 3 hours and analyzed by quantitative real-time PCR (Fig 4A). WT FLSs stimulated with TNFα expressed 4-fold higher levels of S1P_3_ mRNA than untreated WT FLSs. Untreated WT FLSs also had significantly higher levels of S1P_3_ mRNA than S1P_3_-KO FLSs, which were used as a negative control.

### Effect of S1P/S1P_3_ signaling on production of arthritogenic molecules by TNFα-activated FLSs

The role of S1P in TNFα-activated FLSs was investigated. After pretreatment with 10 ng/mL TNFα or vehicle for 8 hours, cells were washed with serum-free DMEM and incubated with 5 μM S1P for the indicated amounts of time (Fig 4B). IL-6 expression was determined by real-time RT-PCR (Fig 4C). IL-6 expression after S1P stimulation was significantly higher in TNFα-primed FLSs than in unprimed FLSs. Furthermore, after TNFα priming, WT FLSs expressed significantly more IL-6 than S1P_3_-KO FLSs. These results were confirmed at the protein level (Fig 4D). When stimulated with S1P, TNFα-primed WT FLSs secreted significantly higher amounts of IL-6 than unprimed WT FLSs or TNFα-primed S1P_3_-KO FLSs (651.6 ± 59.2 pg/mL versus 217.4 ± 26.5 pg/mL or 320.4 ± 13.9 pg/mL respectively).

Levels of MCP-1 and MMP-3 in the same culture supernatant were measured in ELISAs (Fig 4E and 4F). MCP-1 production after S1P stimulation was comparable in WT FLSs and S1P_3_-KO FLSs (2138 ± 948 pg/mL versus 2358 ± 208 pg/mL, p = 0.97), but was increased slightly in WT FLS with TNFα-priming; however, the difference was not statistically significant (2138 ± 948 pg/mL versus 3963 ± 1328 pg/mL, p = 0.58). The differences in MCP-1 levels between the two genotypes were not significant after TNFα-pretreatment and subsequent S1P stimulation (3963 ± 1328 pg/mL versus 3704 ± 1007 pg/mL, p = 0.88). Furthermore, the amounts of MCP-1 secreted by TNFα-primed FLSs were comparable with those with secreted by cells not subsequently stimulated with S1P (WT 4005 ± 1328 pg/mL and S1P_3_-KO 3946 ± 950 pg/mL).

By contrast, MMP-3 production by the two genotypes was comparable after S1P stimulation alone (0.22 ± 0.03 ng/mL versus 0.26 ± 0.02 ng/mL, p = 0.18), and increased significantly in WT FLS with TNFα priming (0.22 ± 0.03 ng/mL versus 5.75 ± 0.18 ng/mL, p < 0.001). When stimulated with S1P after pretreatment with TNFα, WT FLSs secreted significantly more MMP-3 than S1P_3_-KO FLSs (5.75 ± 0.18 ng/mL versus 4.29 ± 0.06 ng/mL, p < 0.001).

## Discussion

The purpose of this study was to investigate the role of S1P_3_ in the CIA mouse model. We demonstrated that S1P_3_ deficiency attenuated both the clinical and histological severity of CIA. While S1P_3_-KO CIA mice showed comparable levels of anti-CII antibodies in serum with WT CIA mice, and comparable expression of TNFα in synovium, they showed significantly lower expression of IL-6 in synovium. Furthermore, in the arthritic synovium, S1P_3_ was upregulated on the FLSs. Primary cultured FLSs showed upregulation of S1P_3_ expression after activation with TNFα, resulting in increased secretion of IL-6 and MMP-3. These results demonstrate that S1P/S1P_3_ signaling promotes the development of CIA and is associated with increased IL-6 secretion by activated FLSs.

S1P and its five receptors have been reported to play roles in several physiological and pathological processes, including bone metabolism, immunity, and cancer [22–24]. Previous studies have demonstrated that S1P levels are increased in RA synovial fluid [7]. In a CIA model, inhibitors and siRNA of sphingosine kinase (SphK), which phosphorylates sphingosine to S1P, suppressed the incidence and the severity of arthritis [25, 26]. Intracellular S1P generated by SphK also functions as an intracellular second messenger, however, it is mainly secreted by its transporter (Spinster 2) and acts an extracellular signaling molecule. Based on previous studies that used mice deficient in SphK1 [27, 28] or Spinster 2 [29], it is suggested that extracellular S1P is more crucial for development of CIA. On the other hand, the role of S1P receptors in the pathogenesis of CIA has not been fully elucidated.

The biological effects of S1P are determined by the expression of its five receptors on target cells. The expression of these receptors is affected by cell differentiation, hypoxia, and cytokine stimulation [23, 24, 30]. A previous study showed that S1P_1_ and S1P_3_ mRNAs are expressed in RA synovia [31] and, in a CIA model, an S1P_1_ antagonist alleviated the severity of CIA [32]. However, it remains unclear whether pharmacological manipulation of S1P/S1P_3_ signaling is effective against inflammatory arthritis. This is the first study to demonstrate that S1P_3_ is upregulated in arthritic synovia and that inhibition of S1P/S1P_3_ signaling alleviates arthritis. We also showed that S1P_3_ expression was upregulated in FLSs activated by TNFα *in vitro*. Taken together, the results suggest that upregulated S1P_3_ on FLSs in the inflamed synovium is associated with CIA severity.

The immunofluorescence staining results showed that PDPN-positive FLSs accumulated in arthritic synovia and that most of them were S1P_3_-positive, whereas FLSs in intact joints were almost all negative for S1P_3_. PDPN on FLSs is upregulated via their activation [10]. These results suggest that S1P_3_ on activated FLSs is upregulated by inflammation and that its increased expression is associated with the severity of CIA. FLSs are activated by several inflammatory factors [33], and activated FLSs play a pivotal role in inflammatory arthritis. FLSs activation is induced by some distinctive features of the arthritic synovium, including hypoxia and abundant cytokines, which leads to further proinflammatory cytokine production, resistance to apoptosis, matrix degradation, and joint destruction [33, 34]. Under conditions of chemical hypoxia, S1P_2_, S1P_3_, and altered S1P metabolism are associated with chemokine production by FLSs [35]; however, expression of these receptors by FLSs is not altered [36]. Since FLSs are also activated by some cytokines [37], we used TNFα as an inducer of FLSs activation and confirmed that S1P_3_ was upregulated in activated FLSs. These data suggest that, in CIA, S1P_3_ is upregulated on FLSs in response to proinflammatory stimuli and its upregulation contributes to the development of the disease.

TNFα also induces IL-6 production in FLSs. It is well recognized that IL-6 plays a key role not only in human RA [38, 39] but also in a murine CIA model [40]. In the present study, IL-6 levels in FLSs were not significantly increased by S1P/S1P_3_ signaling without FLS-activation, but were strongly increased by upregulation of S1P_3_ on FLSs activated with TNFα. Other than IL-6, FLSs release some key mediators of inflammatory arthritis, including MCP-1 and MMPs. Our data show that S1P_3_ deficiency did not affect MCP-1 production by FLSs *in vitro*. Similar to the results for IL-6, the S1P/S1P_3_-induced production of MMP-3 increased significantly upon upregulation of S1P_3_ on FLSs activated by TNFα, although there were modest changes in TNFα-primed FLSs exposed to (or not) subsequent S1P stimulation. These findings indicate that S1P_3_ does not affect the synovium under physiological conditions but exerts its pathogenic function only when the synovium is inflamed. Moreover, our *in vivo* data show that S1P_3_ deficiency decreased expression of IL-6, but not TNFα, in arthritic synovium. Collectively, these results suggest that S1P_3_ contributes to CIA via its ability to upregulate IL-6 production in FLSs activated by TNFα.

Although we demonstrated that S1P_3_ inhibition alleviates experimental arthritis in mice, the underlying mechanism of action is not fully understood. Since S1P_3_ is known to be expressed on other cell types, such as macrophages and lymphocytes, S1P_3_ may contribute to the pathogenesis of inflammatory arthritis via other mechanisms. Considering the contribution of S1P to apoptosis resistance in B cells [7, 41], and to the pathogenicity of B cells and anti-CII antibodies in CIA [42, 43], it was suggested that S1P_3_ expressed by B cells might be associated with development of CIA. Nevertheless, there was no significant reduction in anti-CII antibody levels in S1P_3_-KO CIA mice. It also remains to be determined whether S1P_3_ inhibition can alleviate inflammatory arthritis in humans. Consistent with the previous *in vitro* study of human RA-FLS [8], our study on CIA mice demonstrates that S1P_3_ expressed by CIA FLSs is upregulated by TNFα and associated with IL-6 production, but is not implicated in cell proliferation; by contrast, the effect on MCP-1 is inconsistent among species. It is well-known that IL-6 is a key mediator of inflammatory arthritis and that FLSs are a major source of IL-6 in RA [44]. These findings, along with the results obtained using an animal model, suggest that S1P/S1P_3_ signaling could provide a novel therapeutic target for the treatment of human RA. Further studies should be conducted to investigate other functions of S1P/S1P_3_ signaling in inflammatory arthritis, and to evaluate the efficacy of S1P_3_ inhibition in humans with RA.

## Conclusions

In conclusion, we demonstrated that S1P_3_ deficiency attenuates the severity of CIA and expression of IL-6 in arthritic synovium, and that S1P_3_ is upregulated on FLSs that accumulate in the arthritic synovium. We suggest that alleviation of CIA in S1P_3_-KO mice is associated with reduced IL-6 production from activated FLSs via S1P_3_, which could have potential as a target for the treatment of human RA.

## Supporting information

S1 Appendix Schematic summary of the role of S1P_3_ in CIA FLSs. (PDF)
