# Supplementary material for: Upregulation of sphingosine-1-phosphate receptor 3 on fibroblast-like synoviocytes is associated with the development of collagen-induced arthritis via increased interleukin-6 production
Source: PLoS One. 2019 Jun 7;14(6):e0218090. doi: 10.1371/journal.pone.0218090 (PMC6555509; doi:10.1371/journal.pone.0218090)
Supplement: S1 Appendix — (PDF) [file pone.0218090.s001.pdf]

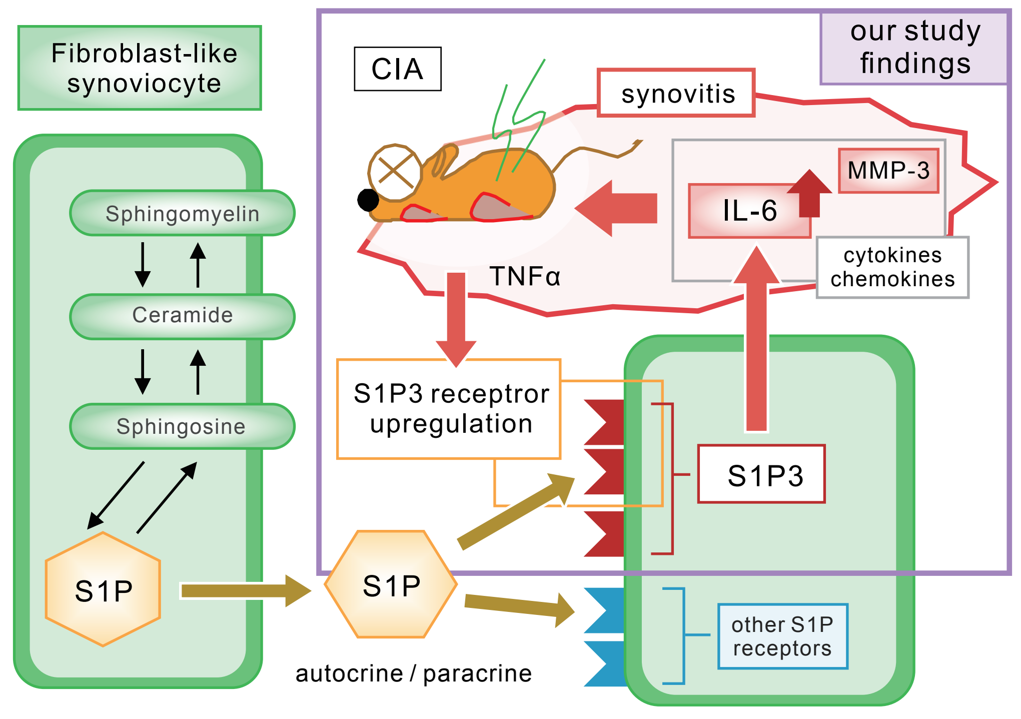

Fibroblast-like synoviocyte
Sphingomyelin
Ceramide
Sphingosine
S1P
S1P
autocrine / paracrine
our study findings
CIA
synovitis
MMP-3
IL-6
cytokines chemokines
TNFα
S1P3 receptror upregulation
S1P3
other S1P receptors
